# Enhancing Communication and Swallowing Skills in Children with Cri Du Chat Syndrome: A Comprehensive Speech Therapy Guide

**DOI:** 10.3390/children11121526

**Published:** 2024-12-16

**Authors:** Soultana Papadopoulou, Areti Anagnostopoulou, Dimitra V. Katsarou, Kalliopi Megari, Efthymia Efthymiou, Alexandros Argyriadis, Georgios Kougioumtzis, Maria Theodoratou, Maria Sofologi, Agathi Argyriadi, Efterpi Pavlidou, Eugenia I. Toki

**Affiliations:** 1 Department of Speech Therapy, School of Health Sciences, University of Ioannina, 45500 Ioannina, Greece; efterpipavlidou@uoi.gr (E.P.); toki@uoi.gr (E.I.T.); 2 Department of Special Education, Hellenic Open University, 26335 Patra, Greece; anagnostopoulou.areti@ac.eap.gr; 3 Department of Preschool Education Sciences and Educational Design, University of the Aegean, 85132 Rhodes, Greece; d.katsarou@aegean.gr; 4 School of Education, University of Nicosia, Nicosia 24005, Cyprus; 5 City College, University of York, Europe Campus, 54622 Thessaloniki, Greece; kmegari@psy.auth.gr; 6 College of Interdisciplinary Studies, Zayed University, Abu Dhabi 144534, United Arab Emirates; efthymia.efthymiou@zu.ac.ae; 7 Department of Nursing, School of Health Sciences, Frederick University, Limassol 3080, Cyprus; hsc.arg@frederick.ac.cy; 8 Department of Turkish Studies and Modern Asian Studies, Faculty of Economic and Political Sciences, National and Kapodistrian, University of Athens, 10559 Athens, Greece; kougioum@turkmas.uoa.gr; 9 Department of Psychology, School of Health Sciences, Neapolis University, Pafos 8042, Cyprus; m.theodoratou@nup.ac.cy; 10 School of Social Sciences, Hellenic Open University, 26335 Patra, Greece; 11 Department of Early Childhood Education, School of Education, University of Ioannina, 45110 Ioannina, Greece; m.sofologi@uoi.gr; 12 Department of Psychology and Social Sciences, Frederick University, Limassol 3080, Cyprus; pre.aa@frederick.ac.cy

**Keywords:** Cri du Chat Syndrome (CdCS), chromosome 5 deletion, speech and language therapy, dysphagia, cognitive impairment

## Abstract

**Background:** A specific deletion on the short arm of chromosome 5 (5p) is the hallmark of the rare genetic syndrome called Cri du Chat Syndrome (CdCS). It causes severe difficulty with swallowing, speech, motor skills, and cognitive deficiencies. These arise from characteristic laryngeal abnormalities and oral–motor dysfunctions. **Objective:** This study aims to investigate the effectiveness of speech and language intervention in addressing the multifaceted challenges of CdCS, including speech and language impairments, feeding difficulties, and social communication deficits. **Methods:** A narrative review was conducted to synthesize existing studies from the last 35 years on therapeutic interventions for individuals with CdCS. This review focused on interventions targeting speech, language, and swallowing therapy. Comprehensive searches were performed in the PubMed and Scopus databases using descriptors such as “Cri du Chat”, “swallowing disorders”, “speech disorders”, “speech and language disorders”, and “speech and language therapy.” From the identified records, 40 peer-reviewed English-language publications that addressed speech, language, and swallowing interventions were selected based on relevance and inclusion criteria. Data extraction was performed independently by four reviewers, working in two teams. Any disagreements between the teams were resolved through discussion with an independent researcher to ensure reliability and minimize bias. **Results:** The findings demonstrate that speech and language therapy (SLT) significantly enhances speech clarity, articulation, and oral–motor coordination. Augmentative communication systems effectively bridge gaps in nonverbal communication, fostering improved social interaction. Specific interventions reduce aspiration risks and improve feeding safety, enhancing the overall quality of life. Early multidisciplinary approaches and tailored therapeutic strategies are key to maximizing the benefits of SLT. **Conclusions:** SLT is crucial for improving communication, swallowing, and social integration in individuals with CdCS. Regular early intervention involving individualized programs and family participation is recommended to achieve optimal outcomes. Further research is needed to evaluate long-term effects and develop cultural and technologically adaptable therapies.

## 1. Introduction

### 1.1. Definition and Clinical Profile

Cri du Chat Syndrome (CdCS) is a rare genetic disorder characterized by a variable severity of symptoms among affected individuals. Early diagnosis, coupled with targeted interventions and supportive therapies, can significantly enhance developmental outcomes and quality of life. Cri du Chat Syndrome (CdCS) poses particular difficulties in diagnosis and treatment. Precisely, it is brought on by a variable-sized deletion on chromosome 5′s short arm (5p-) [1], specifically in the 5p15.3–p15.2 region, which includes the CTNND2 gene critical for neuronal development and connectivity [2,3,4]. Three youngsters with three traits that seemed to form a novel and distinct form of neurodevelopmental impairment were documented by French researchers in 1963 [2]. Along with intellectual incapacity, delayed speech, and dysmorphic characteristics, the disease is known for its characteristic “cat-like” cry, which is caused by structural abnormalities in the larynx [5,6].

A defining feature of CdCS, which inspired its name, is the unique, cat-like cry observed in affected infants [7]. This cry has been characterized as “a distinct high-pitched, monochromatic sound at birth”, resembling the mewing of a cat [8,9]. While this distinctive cry is commonly associated with the syndrome, it is essential to note that it may not be present in every case. Nevertheless, its high prevalence and rarity in other disorders make it a valuable and early diagnostic marker for CdCS [7].

### 1.2. Epidemiology

Understanding the clinical manifestations of CdCS is crucial, but equally important is recognizing the epidemiological aspects of this syndrome, which provide insight into its prevalence and demographic distribution. Despite being characterized as a rare genetic autosomal syndrome, CdCS is a human’s frequent chromosomal syndrome. The incidence has been reported to range from approximately 1 in 15,000 to 1 in 50,000 live births, with no significant difference observed in the male-to-female ratio [10]. Subsequent epidemiological studies have revised this estimate to a more precise frequency of 1 in 37,000 live births [11]. CdCS demonstrates a slightly higher prevalence in females, with a reported male-to-female ratio of 3:4 [12]. Studies have found no significant variation in the prevalence of CdCS across different racial or geographical populations [11]. Additionally, no strong associations have been established with prenatal events, parental age, or birth order [13,14].

Counseling on genetic issues is critical for families affected by CdCS, mainly because female patients retain fertility and have the potential to pass the syndrome to their offspring. In cases where a parent carries a structural chromosomal rearrangement, the recurrence risk for CdCS in their children is estimated at 50% [14,15]. Although CdCS generally presents with significant cognitive and developmental challenges, recent studies indicate that some individuals may experience relatively mild cognitive impairment despite the genetic deletion [16,17].

### 1.3. Etiology

CdCS is a genetic condition that is predominantly non-hereditary. Approximately 10% of individuals with CdCS inherit the chromosomal abnormality from an asymptomatic parent [18]. In 10–15% of cases, the condition arises from parental chromosomal translocations [1,19], and fewer than 10% are linked to rare cytogenetic aberrations [14]. In such cases, the parent carries a balanced translocation, a chromosomal rearrangement where no genetic material is lost or gained. Although balanced translocations typically do not cause health issues in the carrier, they may result in unbalanced rearrangements when transmitted to offsprings, leading to genetic disorders such as CdCS [18].

Most CdCS cases result from a de novo deletion on the short arm of chromosome 5 (5p), meaning the genetic alteration occurs spontaneously and is not inherited. This chromosomal deletion, first identified by geneticist Jérôme Lejeune in 1963, was linked to craniofacial malformations and developmental delays [2]. Approximately 85% of CdCS cases are attributed to these de novo deletions, with the syndrome also referred to as “5p minus” syndrome [20].

The chromosomal deletions associated with CdCS occur with an estimated incidence of 1 in 50,000 live births. These deletions can be terminal (78%), interstitial (9%), or result from unbalanced translocations (5%). Deletions in the 5p15.2 region are implicated in the characteristic dysmorphism and intellectual disability, while deletions in 5p15.3 are associated with the distinctive cat-like cry and speech delays. Molecular studies have refined the understanding of these regions, identifying specific genes responsible for various syndrome features [21].

Phenotypic variability in CdCS is largely influenced by the size of the chromosomal deletion. Cerruti Mainardi et al. (2001) [16] demonstrated that larger deletions are associated with more severe psychomotor delays. This size-dependent variability affects the degree of intellectual and developmental impairment, as the loss of multiple genes on 5p contributes to clinical presentation. For instance, the CTNND2 gene has explicitly been linked to severe intellectual disability in some patients with CdCS [18]. This comprehensive understanding of the genetic and molecular basis of CdCS highlights the importance of chromosomal region 5p15 in the pathognomonic features of the condition, including its cognitive, developmental, and physical manifestations.

Despite progress in understanding CdCS, significant gaps remain in optimizing therapeutic approaches. While speech and language therapy (SLT) has shown promise in improving communication, swallowing, and oral–motor coordination, comprehensive studies on its long-term efficacy and tailored interventions for the syndrome’s variability are lacking [22]. Further exploration is needed regarding the integration of interdisciplinary methods, age-specific therapies, and technology-driven tools like augmentative and alternative communication (AAC) devices for nonverbal children [23]. More research is needed on cultural and demographic factors affecting therapy outcomes and the psychosocial impacts on caregivers and families [24]. Addressing these gaps could enhance the effectiveness of therapeutic interventions and improve the quality of life for individuals with CdCS and their families.

This study aims to comprehensively explore the clinical picture of CdCS and its associated challenges, focusing on SLT as a therapeutic choice. It seeks to describe the spectrum of communication, eating and swallowing difficulties, oral–motor impairments, and social communication challenges observed in CdCS, while examining the strategies employed in SLT to address these issues. The research emphasizes the role of SLT in managing these multifaceted challenges, offering insights into current practices and potential therapeutic advancements which could improve functional outcomes and quality of life for individuals with CdCS.

## 2. Methods

The research question of this study was the following: “What are the key clinical challenges in communication, eating, and swallowing among individuals with CdCS, and how can SLT strategies effectively address these issues to enhance functional outcomes and quality of life?”

### 2.1. Search Strategy

A comprehensive exploration of scholarly databases, including PubMed and Scopus, was conducted to identify relevant articles published between 1989 and 2024. The search terms used included combinations such as the following:

- “cri du chat AND swallowing disorders” (PubMed: 3 results; Scopus: 2 results);
- “cri du chat AND speech disorders” (PubMed: 21 results; Scopus: 35 results);
- “cri du chat AND speech and language disorders” (PubMed: 17 results; Scopus: 35 results);
- “cri du chat AND speech and language therapy” (PubMed: 8 results; Scopus: 8 results).

Searches in Scopus used Boolean connectors (e.g., “cri AND du AND chat AND swallowing AND disorders”) to refine the results. These searches aimed to identify studies exploring swallowing, speech, and language disorders, particularly in CdCS.

### 2.2. Inclusion Criteria

Studies were included based on the following criteria:

- Regarding language, articles in English for a pediatric population diagnosed with CdCS were included.
- Regarding content, articles describing clinical characteristics and interventions with SLT programs for rehabilitating speech, communication, and swallowing disorders were included.
- Regarding study type, clinical trials, observational studies, case studies, and other relevant research, peer-reviewed articles, and book chapters focusing on swallowing, speech, and language disorders or therapies associated with CdCS were included, but studies were prioritized if they addressed speech and language interventions or specific swallowing issues.
- Regarding publication date, studies published within the last 35 years were included.

### 2.3. Exclusion Criteria

The following were excluded: non-English publications, non-peer-reviewed studies, articles without full-text availability, and studies that did not directly address the targeted disorders in CdCS.

### 2.4. Study Selection

The initial search yielded a total of 129 results across PubMed and Scopus. After eliminating 37 duplicates and conducting title and abstract screening, 92 articles were identified as potentially relevant. These were further reviewed in full text, resulting in a final selection of 40 articles based on their relevance to the research questions and the quality of their study design.

### 2.5. Data Extraction

Data were systematically extracted from the selected articles, focusing on the following:

- Study objectives and methodologies;
- Key findings on swallowing, speech, and language disorders;
- Therapeutic approaches and their effectiveness;
- Future recommendations for research in this domain.

The extracted data were organized in a tabular format for comparative analysis and thematic synthesis.

### 2.6. Quality Assessment

Due to the narrative nature of this review, a formal quality assessment tool was not utilized. However, each study was critically evaluated based on the following:

- Methodological rigor;
- Sample size;
- Relevance to the research intentions;
- Clarity and reliability of the findings.

### 2.7. Synthesis

The synthesis required organizing the literature into key thematic categories, including the following:

1. Clinical characteristics of individuals with CdCS;
2. Speech, language, and swallowing difficulties;
3. Speech, language, and swallowing intervention proposals.

The findings were presented narratively, highlighting patterns, gaps, and areas for future exploration.

## 3. Results

Table 1 displays the studies included in this narrative review used to synthesize the existing literature on therapeutic interventions targeting speech, language, and swallowing rehabilitation of individuals with CdCS.

**Table 1:** Studies included in the narrative review.

Reference	Authors	Title–DOI	Year
[1]	Sigafoos, J.; O’Reilly, M.F.; Lancioni, G.E.	Cri-Du-Chat. Developmental Neurorehabilitation doi:10.1080/17518420902975720	2009
[4]	Holland, P.; Wildhagen, M.; Istre, M.; Reiakvam, O.M.; Dahl, J.A.; Søraas, A.	Cri Du Chat Syndrome Patients Have DNA Methylation Changes in Genes Linked to Symptoms of the Disease doi:10.1186/S13148-022-01350-3	2022
[7]	Cornish, K.; Bramble, D.	Cri Du Chat Syndrome: Genotype-Phenotype Correlations and Recommendations for Clinical Management doi:10.1017/S0012162201002419	2002
[9]	Mainardi, P.C.; Pastore, G.; Castronovo, C.; Godi, M.; Guala, A.; Tamiazzo, S.; Provera, S.; Pierluigi, M.; Bricarelli, F.D.	The Natural History of Cri Du Chat Syndrome. A Report from the Italian Register doi:10.1016/J.EJMG.2005.12.004	2006
[10]	Pizzamiglio, M.R.; Volpe, C.; Piccardi, L.; Pizzamiglio, M.R.; Volpe, C.; Piccardi, L.A.	Longitudinal Study in Atypical Cri-Du Chat Profile: A Single Case Report doi:10.4236/CRCM.2013.22027	2013
[12]	Bagchi, N.R.; Bhanja, S.	Cri Du Chat Syndrome: A Case Report with Recurrent Pneumonia and Chronic Stridor doi:10.22038/IJP.2015.5143	2015
[14]	Rodríguez-Caballero, Á.; Torres-Lagares, D.; Rodríguez-Pérez, A.; Serrera-Figallo, M.Á.; Hernández-Guisado, J.M.; Machuca-Portillo, G.	Cri Du Chat Syndrome: A Critical Review doi:10.4317/MEDORAL.15.E473	2010
[15]	Didden, R.; Curfs, L.	Cri Du Chat Syndrome doi:10.1016/B978-0-12-374984-0.00353-3	2013
[20]	Sweeney, S.	Cri Du Chat Syndrome: Case Presentation and Review	2012
[21]	Espirito Santo, L.D.; Moreira, L.M.A.; Riegel, M.	Cri-Du-Chat Syndrome: Clinical Profile and Chromosomal Microarray Analysis in Six Patients doi:10.1155/2016/5467083.	2016
[23]	Cornish, K.M.; Pigram, J.	Developmental and Behavioural Characteristics of Cri Du Chat Syndrome. Archives of disease in childhood doi:10.1136/ADC.75.5.448	1996
[25]	Tullu, M.S.; Muranjan, M.N.; Sharma, S.V.; Sahu, D.R.; Swami, S.R.; Deshmukh, C.T.; Bharucha, B.A.	Cri-Du-Chat Syndrome: Clinical Profile and Prenatal Diagnosis	1998
[26]	Cornish, K.M.; Munir, F.	Receptive and Expressive Language Skills in Children with Cri-Du-Chat Syndrome doi:10.1016/S0021-9924(97)00052-X	1998
[27]	Kristoffersen, K.E.	Speech and Language Development in Cri Du Chat Syndrome: A Critical Review doi:10.1080/02699200801892108	2008
[28]	Buggenhout, G.; Pijkels, E.; Holvoet, M.; Schaap, C.; Hamel, B.; Fryns, J.	Cri Du Chat Syndrome: Changing Phenotype in Older Patients doi:10.1002/(SICI)1096-8628(20000131)90:3	2000
[29]	Cornish, K.M.	The Neuropsychological Profile of Cri Du Chat Syndrome without Significant Learning Disability doi:10.1111/J.1469-8749.1996.TB15050.X	1996
[30]	Liverani, M.E.; Spano, A.; Danesino, C.; Malacarne, M.; Cavani, S.; Spunton, M.; Guala, A.	Children and Adults Affected by Cri Du Chat Syndrome: Care’s Recommendations doi:10.4081/PR.2019.7839	2019
[31]	Guala, A.; Spunton, M.; Kalantari, S.; Kennerknecht, I.; Danesino, C.	Neoplasia in Cri Du Chat Syndrome from Italian and German Databases doi:10.1155/2017/5181624	2017
[32]	Lokmanoğlu, B.N.Y.; Mutlu, A.; Livanelioğlu, A.; Haliloğlu, G.	The General Movements Assessment and Effects of an Early Intervention in an Infant with Cri Du Chat Syndrome: A Case Report doi:10.24953/TURKJPED.2021.01.021	2021
[33]	Virbalas, J.M.; Palma, G.; Tan, M.	Obstacles to Communication in Children with Cri Du Chat Syndrome doi:10.1016/J.JVOICE.2012.06.005	2012
[34]	Honjo, R.S.; Mello, C.B.; Pimenta, L.S.E.; Nuñes-Vaca, E.C.; Benedetto, L.M.; Khoury, R.B.F.; Befi-Lopes, D.M.; Kim, C.A.	Cri Du Chat Syndrome: Characteristics of 73 Brazilian Patients doi:10.1111/JIR.12476	2018
[35]	Butt, A.K.; Zubair, R.; Rathore, F.A.	The Role of Augmentative and Alternative Communication in Speech and Language Therapy: A Mini Review doi:10.47391/JPMA.22-023	2022
[36]	Sofologi, M.; Papatzikis, E.; Kougioumtzis, G.; Kosmidou, E.; Klitsioti, A.; Droutme, A.; Sourbi, A.A.; Chrisostomou, D.; Efstratopoulou, M.	Effectiveness of Musical Training on Reading Comprehension in Elementary School Children. Is There an Associative Cognitive Benefit? doi:10.3389/FEDUC.2022.875511/BIBTEX	2022
[37]	Giridhar, S.	Cri-Du-Chat Syndrome: A Case Report	2020
[38]	Taheri, A.; Meghdari, A.; Alemi, M.; Pouretemad, H.; Poorgoldooz, P.; Roohbakhsh, M.	Social Robots and Teaching Music to Autistic Children: Myth or Reality? doi:10.1007/978-3-319-47437-3_53	2016
[39]	Triggs, J.; Pandolfino, J.	Recent Advances in Dysphagia Management doi:10.12688/F1000RESEARCH.18900.1/DOI	2019
[40]	Cornish, K.M.; Bramble, D.; Munir, F.; Pigram, J.	Cognitive Functioning in Children with Typical Cri Du Chat (5p-) Syndrome doi:10.1017/S0012162299000559	1999
[41]	Levy, B.; Dunn, T.M.; Kern, J.H.; Hirschhorn, K.; Kardon, N.B.	Delineation of the Dup5q Phenotype by Molecular Cytogenetic Analysis in a Patient with Dup5q/Del 5p (Cri Du Chat) doi:10.1002/AJMG.10261	2002
[42]	Marinescu, R.C.; Johnson, E.I.; Grady, D.; Chen, X.N.; Overhauser, J.	FISH Analysis of Terminal Deletions in Patients Diagnosed with Cri-Du-Chat Syndrome doi:10.1034/J.1399-0004.1999.560405.X	1999
[43]	Lass, N.J.; Pannbacker, M.	The Application of Evidence-Based Practice to Nonspeech Oral Motor Treatments doi:10.1044/0161-1461(2008/038)	2008
[44]	Nakao-Kato, M.; Rathore, F.A.	An Overview Of The Management And Rehabilitation Of Dysphagia doi:10.47391/JPMA.23-61	2023
[45]	Zhang, X., Snijders, A., Segraves, R., Zhang, X., Niebuhr, A., Albertson, D., … & Pinkel, D.	High-resolution mapping of genotype–phenotype relationships in cri du chat syndrome using array comparative genomic hybridization doi: 10.1086/427762	2005
[46]	Wu, Q.; Niebuhr, E.; Yang, H.; Hansen, L.	Determination of the ‘critical region’ for cat-like cry of Cri-du-chat syndrome and analysis of candidate genes by quantitative PCR doi:10.1038/sj.ejhg.5201345	2005
[47]	Castillo, J.C.; Álvarez-Fernández, D.; Alonso-Martín, F.; Marques-Villarroya, S.; Salichs, M.A.	Social Robotics in Therapy of Apraxia of Speech doi:10.1016/J.EJPN.2020.07.002	2018
[48]	Villa, R.; Fergnani, V.G.C.; Silipigni, R.; Guerneri, S.; Cinnante, C.; Guala, A.; Danesino, C.; Scola, E.; Conte, G.; Fumagalli, M.; et al.	Structural Brain Anomalies in Cri-Du-Chat Syndrome: MRI Findings in 14 Patients and Possible Genotype-Phenotype Correlations doi:10.1016/J.EJPN.2020.07.002	2020
[49]	Sanders, C.D.; Leigh, M.W.; Chao, K.C.; Weck, K.E.; King, I.; Wolf, W.E.; Campbell, D.J.; Knowles, M.R.; Zariwala, M.A.; Shapiro, A.J.	The Prevalence of the Defining Features of Primary Ciliary Dyskinesia within a Cri Du Chat Syndrome Cohort doi:10.1002/PPUL.24159	2018
[50]	Bel-Fenellós, C.; Biencinto-López, C.; Sáenz-Rico, B.; Hernández, A.; Sandoval-Talamantes, A.K.; Tenorio-Castaño, J.; Lapunzina, P.; Nevado, J.	Cognitive-Behavioral Profile in Pediatric Patients with Syndrome 5p-; Genotype-Phenotype Correlationships. doi:10.3390/GENES14081628	2023
[51]	Wapner, R.J.; Babiarz, J.E.; Levy, B.; Stosic, M.; Zimmermann, B.; Sigurjonsson, S.; Wayham, N.; Ryan, A.; Banjevic, M.; Lacroute, P.; et al.	Expanding the Scope of Noninvasive Prenatal Testing: Detection of Fetal Microdeletion doi:10.1016/J.AJOG.2014.11.041	2015
[52]	Ye, Y.; Luo, Y.; Qian, Y.; Xu, C.; Jin, F.	Cri Du Chat Syndrome after Preimplantation Genetic Diagnosis for Reciprocal Translocation doi:10.1016/J.FERTNSTERT.2011.04.080	2011
[53]	Georgieva-Tsaneva, G.; Andreeva, A.; Tsvetkova, P.; Lekova, A.; Simonska, M.; Stancheva-Popkostadinova, V.; Dimitrov, G.; Rasheva-Yordanova, K.; Kostadinova, I.	Exploring the Potential of Social Robots for Speech and Language Therapy: A Review and Analysis of Interactive Scenarios doi:10.3390/machines11070693	2023

### 3.1. Clinical Characteristics of Individuals with CdCS

The area of the chromosome that has been eliminated and whether it is terminal or interstitial affect the syndrome’s clinical manifestation, severity, and course. In other words, variations in genotype can be linked to variations in phenotype [15]. Individuals with CdCS frequently exhibit the condition’s hallmarks, such as craniofacial abnormalities and the high-pitched, monotonous, cat-like cry which is nearly always heard in infants. Individuals with CdCS usually have telecanthus and epicanthal folds. There has also been evidence of an increased risk of strabismus [20].

Clinical characteristics encompass microcephaly, internal deformities, certain facial dysmorphias, and anomalies of the larynx that result in vocal dysfunctions. Severe mental and psychomotor impairment is a hallmark of cognitive development [25].

Numerous studies have identified a triad of core features in individuals with CdCS: psychomotor delay, limited verbal abilities, and significant learning challenges. Importantly, not all affected children exhibit profound intellectual disabilities; some demonstrate cognitive abilities nearing the typical range. This variability underscores the necessity of conducting comprehensive assessments of each child’s intellectual and cognitive functioning. Beyond intellectual impairments, children with CdCS often experience pronounced deficits in learning abilities, language acquisition, communication, academic performance, and adaptive behavior skills [1]. Cornish et al. (1996) reported severe language delays, characterized by a marked discrepancy between relatively preserved comprehension and profoundly impaired speech production [23]. Verbal development progresses notably slowly, with receptive language abilities generally surpassing expressive capabilities. Some individuals may achieve success using alternative communication methods, such as sign language, to mitigate these challenges [14].

Behavioral challenges are frequently observed in children with CdCS, including severe self-injurious behaviors, aggression, and stereotypic movement disorders [1]. Self-injurious behaviors commonly manifest in three patterns: striking the head against objects, hitting the head with body parts, and self-inflicted injuries through biting. Additional behavioral features include hypersensitivity to auditory stimuli, repetitive movements, clumsiness, excessive euphoria, stubbornness, compulsive attachment to specific objects, and echolalia. Early intervention strategies such as occupational therapy, psychological support, and parental counseling have effectively mitigated these issues [14].

A hallmark feature of CdCS is the presence of various physical anomalies. These include microcephaly, hypertelorism, a rounded, moon-shaped facial appearance, low-set ears, micrognathia, and other craniofacial dysmorphisms [1,15]. At birth, the clinical features are more detailed, with everyday observations including a low birth weight (mean: 2614 g), microcephaly (mean head circumference: 31.8 cm), rounded facial features (83.5%), a broad nasal bridge (87.2%), hypertelorism (81.4%), epicanthal folds (90.2%), downward-slanting palpebral fissures (56.9%), downward-turned corners of the mouth (81.0%), low-set ears (69.8%), micrognathia (96.7%), and abnormal dermatoglyphics such as transverse flexion creases (92%). The distinctive cat-like cry, characteristic of CdCS, is reported in 95.9% of cases, according to the Italian CdCS Registry. Neonatal complications often include perinatal asphyxia, cyanotic episodes, impaired sucking ability, and hypotonia. Severe psychomotor delays become evident within the first year of life. Congenital malformations, though less frequent, may affect the cardiac, neurological, and renal systems and include features such as preauricular tags, syndactyly, hypospadias, and cryptorchidism. Recurrent respiratory and intestinal infections are prevalent during the early years, likely attributable to swallowing dysfunction, aspiration, and impaired muscular coordination; however, increased susceptibility to infections is not a general feature of CdCS [9]. During the first two years of a child’s life, significant developmental milestones occur that shape their physical, cognitive, and emotional growth, primarily due to feeding difficulties. These challenges stem from impaired swallowing coordination, aspiration, dysphagia, muscle hypotonia, and gastroesophageal or nasal reflux, all of which can severely compromise nutritional intake and growth. As a result, dysphagia becomes a critical clinical issue, significantly influencing the overall developmental outcomes during this crucial period [14].

The following craniofacial dysmorphic features are commonly observed in individuals with CdCS: an elongated and narrow face (70.8%), a prominent supra-orbital ridge (31.0%), a shortened philtrum (87.8%), a full lower lip (45.2%), dental malocclusion such as an open bite (75.0%), horizontally oriented palpebral fissures (70.2%), frequent divergent strabismus (44.7%), and shortened metacarpals (82.6%) and metatarsals (75.0%), resulting in small hands and feet. Additionally, approximately 30.4% of patients may present with gray hair, as reported by the Italian CdCS Registry. Over time, muscle hypotonia typically transitions to hypertonia, and microcephaly becomes more pronounced. Seizures are rare across all age groups. Neuroimaging studies have revealed brainstem atrophy, particularly involving the pons, cerebellum, median cerebellar peduncles, and cerebellar white matter. An isolated case of a child with an arachnoid cyst causing triventricular hydrocephalus due to aqueductal stenosis has been documented, though such findings are atypical of CdCS. Metabolic abnormalities have been reported, including defects in purine nucleotide synthesis—neuromodulators essential for brain development. Additional cases have described clinical features associated with non-ketotic hyperglycinemia, infantile spasms, hypsarrhythmia, and brain heterotopia in patients with a 5p deletion, consistent with CdCS. Cryptorchidism, while occasionally present at birth, is uncommon in adolescent patients, and sexual development is generally expected in both sexes. Reports of myopia and cataracts are also noted [9].

Other frequently observed features include a downturned mouth with excessive salivary drooling due to orofacial muscle hypotonia, low-set ears, a short stature, single palmar creases, and congenital cardiac defects such as ventricular septal defects, atrial septal defects, patent ductus arteriosus, and tetralogy of Fallot [12]. Behavioral characteristics often include attention deficit disorders with hyperactivity, present in approximately 50% of cases, which may co-occur alongside aggression [23]. These behaviors can be mitigated through tailored therapeutic interventions. Despite these challenges, children with CdCS are typically described as gentle and affectionate. Only a small number of cases have exhibited autistic traits or significant social interaction impairments [14].

Table 2 outlines the key clinical attributes of CdCS, emphasizing the incidence and impact of various features.

#### 3.1.1. Speech, Language, and Swallowing Difficulties in CdCS

Speech and language impairments are a hallmark feature of CdCS, often manifesting as one of the most significant neurocognitive challenges for affected individuals. These impairments typically involve both expressive and receptive language, with expressive abilities being more severely compromised. Children with CdCS generally experience substantial delays in speech production. Many individuals remain nonverbal or have severely limited verbal output throughout their lives [23]. Those who develop speech may accomplish so at a slower rate, with articulation difficulties and limited vocabulary. The average age of first spoken words is often delayed compared to typical developmental milestones, and some children may not achieve functional speech, relying on alternative means of communication such as gestures, picture communication systems, or sign language [23]. Limited verbal output is frequently associated with poor phonological memory, which affects the ability to form and recall words [23]. Although receptive language abilities are generally more advanced than expressive skills, they are still impaired in most individuals with CdCS. Children with CdCS may understand simple instructions and basic vocabulary but struggle with more complex language, such as abstract concepts or multi-step directions. This discrepancy between receptive and expressive abilities suggests that, while some language comprehension is present, the capacity to articulate thoughts remains limited due to motor and cognitive constraints [9].

In examining the neurocognitive and linguistic characteristics of individuals with CdCS, one notable feature is a delay in the onset of spoken words. Research indicates that, on average, children with CdCS begin to produce their first spoken words between 2.5 and 5 years of age, which is significantly delayed compared to the typical developmental milestone of around 12 months [7,26]. This delay can be attributed to cognitive, motor, and linguistic factors impacting speech development. In addition to delayed expressive language, children with CdCS often experience difficulties in receptive language skills, further compounding communication challenges [9]. Understanding these developmental timelines provides valuable context for assessing and supporting language acquisition in this population.

Several factors contribute to the severity of speech and language impairments in CdCS, such as hypotonia, cognitive limitations, and oral–motor difficulties. A low muscle tone, or hypotonia, particularly in the muscles used for speech, is a common physical characteristic of CdCS. This affects the motor coordination required for articulation and can contribute to dysarthria (speech sound disorders caused by muscle weakness) [9]. Intellectual disability, which is prevalent in CdCS, has a significant meaning regarding the delay and impairment of language skills. Cognitive deficits, particularly in working memory and attention, hinder learning and applying language rules effectively [23]. Beyond hypotonia, oral–motor dysfunctions, including difficulties with tongue movement and other oral structures, contribute to articulation and speech clarity challenges. These difficulties can lead to more severe speech production issues. Feeding and swallowing difficulties are usual findings in infants with CdCS. These challenges include sucking problems caused by muscle weakness and difficulty latching onto the nipple due to impaired motor control of feeding-related muscles. Additionally, frequent gastroesophageal reflux occurs due to poor coordination and weakness of the swallowing muscles. Hypotonia often leads to an inability to manage saliva, a delayed initiation of the swallowing reflex, and ineffective bolus control due to reduced tongue movement. Consequently, swallowing becomes unsafe, increasing the risks of malnutrition, dehydration, and potentially life-threatening aspiration [27].

Executive functioning and attention deficits are core neurocognitive challenges in individuals with CdCS. These deficits impact problem-solving, goal-directed behavior, attention regulation, and other higher-order cognitive processes necessary for daily functioning. Executive functioning encompasses vital cognitive processes like planning, working memory, flexible thinking, and impulse control. In (CdCS), the genetic deletion on chromosome 5p represents an additional challenge associated with brain neuroplasticity and its developmental potential [7].

Foundational research has identified deficits in early attentional mechanisms as a central feature of CdCS, with difficulties in sustained attention and attentional shifting observed in affected individuals. These attentional impairments hinder cognitive processing and serve as a foundation for further deficits in executive functioning, such as inhibitory control and working memory, which are critical for suppressing irrelevant stimuli and temporarily holding information necessary for complex tasks [7]. Linguistically, CdCS is marked by delays in expressive and receptive language abilities, partly due to these early attentional and memory-related challenges [29].

Working memory, which refers to the capacity to hold and manipulate information over brief periods, is often compromised in individuals with CdCS. This impairment can significantly impact their ability to process and utilize information effectively in daily tasks. This can lead to difficulties in performing tasks requiring tracking multiple steps or instructions [23]. Individuals with CdCS may struggle with remembering the information necessary for completing tasks, leading to frequent errors and the need for repetition.

Beyond these core deficits, other memory systems—including long-term and declarative memory—also display variability, with some individuals experiencing notable challenges in encoding, storing, and retrieving information over time. These memory difficulties further impact language acquisition and the ability to apply learned concepts in daily life, which complicates adaptive functioning [7].

The neurocognitive profile of CdCS thus reflects a broad yet interconnected array of challenges spanning attention, executive function, and multiple memory systems, underscoring the need for tailored clinical interventions which focus on enhancing early attentional skills, reinforcing executive functions, and supporting language development. Such personalized approaches can better support individuals with CdCS in reaching their cognitive and communicative potential, addressing both neurodevelopmental and adaptive needs.

Planning and problem-solving are also significantly affected. Children with CdCS often have difficulty organizing thoughts and actions, especially in complex situations. They may exhibit challenges in breaking down tasks into manageable parts or foreseeing the consequences of their actions, making it hard to achieve long-term goals or adapt to new situations [7]. Cognitive flexibility, the ability to shift thinking or adapt to new information, is also impaired in CdCS. This makes it difficult for affected individuals to adjust to changes in routines or expectations. They may exhibit rigid, repetitive behaviors and show resistance to change [9]. Inhibitory control, which allows individuals to suppress inappropriate responses, is another area of concern. Children with CdCS may have difficulty controlling impulses, leading to inappropriate or impulsive behaviors. This lack of impulse control can also contribute to social and behavioral challenges, as individuals may struggle to regulate their actions in social settings [23].

Attention deficits are prevalent in individuals with CdCS, often exacerbating executive functioning difficulties. Children with CdCS may have difficulty maintaining attention on tasks for extended periods. This problem with sustained attention can lead to poor task performance and difficulties in school or structured activities that require focus [23]. As a result, they may exhibit behaviors similar to the attention deficit hyperactivity disorder (ADHD), with symptoms of inattention, distractibility, and hyperactivity. Selective attention, or the ability to focus on relevant stimuli while ignoring distractions, is also impaired. Children with CdCS often struggle to filter out extraneous stimuli, making it hard for them to concentrate in noisy or stimulating environments [9]. This can lead to frustration and difficulty completing tasks, as they may be easily sidetracked by surrounding activities or sensory inputs. Attention-shifting, the ability to switch focus from one task or stimulus to another, is another challenge for children with CdCS. This is closely related to cognitive flexibility and can result in difficulty transitioning between activities or adapting to new tasks. These difficulties may lead to frustration, behavioral outbursts, or resistance to changes in routine [9].

Memory impairments are a significant aspect of the neurocognitive profile in individuals with CdCS. These impairments affect both working and long-term memory, contributing to broader cognitive difficulties, including learning, problem-solving, and language development challenges. Working memory is defined as the cognitive capability to retain and process information over brief intervals. In individuals with CdCS, working memory is notably impaired, which has widespread implications for daily functioning. Studies show that children with CdCS have particular difficulties with verbal working memory tasks, which involve retaining and processing spoken information. This may contribute to the already significant language delays seen in the syndrome, as individuals struggle to retain phonological or syntactic information during conversation or instruction. For instance, children may have trouble following multi-step instructions or remembering sequences of tasks, which can negatively affect academic achievement and the ability to engage in structured activities [26]. Additionally, the impairment in verbal working memory is compounded by the overall intellectual disability present in most individuals with CdCS, making it difficult for them to apply cognitive strategies to compensate for memory deficits [9].

Although long-term memory, mainly nonverbal memory, appears to be relatively less affected than working memory in individuals with CdCS, deficits in this domain remain evident. Studies suggest that episodic memory, defined as the ability to retrieve personal experiences and specific events, is notably compromised. Such impairments can significantly affect the capacity to recall critical details from past events and encode, retain, and retrieve newly acquired information [29]. This is particularly evident in recalling factual or learned information, which is necessary for academic tasks such as remembering historical dates, or mathematical procedures.

Declarative memory, which involves recalling facts and events, can also be impaired. Some children with CdCS may have difficulty learning and recalling basic concepts, such as colors, shapes, or the names of familiar people and places, which affects their ability to engage in social and academic environments [23]. Interestingly, nonverbal memory tasks, particularly those involving visual–spatial memory, are relatively stronger in some individuals with CdCS. This relative strength in visual–spatial abilities can be leveraged to support learning and memory in other domains. For example, individuals may benefit from visual aids or pictorial learning methods that rely on their better-developed ability to recall images and spatial relationships [23]. This is particularly useful in educational settings, where visual supports can help reinforce verbal instructions or abstract concepts. However, even within visual memory, the complexity of the task plays a role. More complex visual–spatial tasks, which require the integration of multiple pieces of information over time, may still be challenging for individuals with CdCS, especially when combined with attention and executive functioning deficits [9].

The memory impairments in CdCS profoundly affect learning, social interactions, and independence. In a learning environment, retaining new information can be challenging, impacting the development of academic skills such as reading, writing, and mathematics. Children may require constant repetition and visual cues to help encode information into memory. This can slow down learning progress and necessitate specialized teaching strategies tailored to their cognitive profile [7]. In daily life, memory impairments also impact functional independence. Individuals with CdCS may forget routines, important events, or personal information, making it challenging to manage day-to-day tasks without external support. Memory deficits can also affect social relationships, as individuals may struggle to remember conversations, names, or past interactions with others, leading to social withdrawal or misunderstandings [23].

Early and consistent interventions are critical in helping individuals with CdCS cope with memory impairments. Cognitive interventions emphasizing repetition, visual aids, and multi-sensory learning can help compensate for verbal memory deficits. Teaching strategies that use concrete visual examples and frequent practice are recommended in educational contexts. Moreover, memory training exercises that strengthen working memory and cognitive flexibility, such as memory games or interactive software, may also benefit children with CdCS [9].

Language and memory impairments in CdCS, particularly in speech, verbal working memory, and long-term episodic memory, play a crucial role in the cognitive difficulties experienced by affected individuals. These deficits affect learning, social interactions, and everyday functioning but can be managed to some extent with appropriate interventions and support strategies.

#### 3.1.2. Diagnosis and Differential Diagnosis

The diagnosis of CdCS is primarily clinical, relying on characteristic features such as distinct facial dysmorphisms (facial gestalt), transverse flexion creases, hypotonia, and the hallmark cat-like cry. The initial diagnostic procedure involves conventional karyotype analysis, which serves to confirm the chromosomal abnormality. In cases where clinical suspicion persists despite a typical karyotype result, fluorescence in situ hybridization (FISH) analysis is recommended. This technique enables the precise visualization and mapping of genetic material within cells, including specific genes or gene regions, thereby resolving diagnostic ambiguities and providing greater molecular clarity [9,41,42].

After chorionic villus sampling during amniocentesis, a diagnosis of the deletion of the short arm of chromosome 5 may happen by chance; typically, this is performed in light of the mother’s age or response to abnormal ultrasound results. Apart from traditional cytogenetics and comparative genomic hybridization (CGH array), fetal DNA analysis on maternal blood is the latest technique to detect the deletion. It makes sense to store biological material in a biobank once a diagnosis has been made. In neonates, the diagnosis is typically indicated by a constellation of clinical features, including the distinctive high-pitched, cat-like cry, low birth weight, microcephaly, and characteristic facial dysmorphisms [30].

Performing a thorough cardiologic evaluation using an ECG and ultrasound is recommended to rule out any cardiac malformations. Studies have estimated that 15% to 20% of patients with CdCS syndrome have cardiac anomalies, though the precise incidence of these conditions is still unknown. This percentage increases to 55% in patients with an unbalanced translocation, and it is linked to more complicated diagnoses like endocardial cushion defect and Fallot tetralogy. In the initial evaluation, pulse oximetry can help keep an eye on the patient during the first few days. Like all newborns, the infant will undergo testing for otoacoustic emissions and ocular red reflexes [31].

Metabolic screening, currently available to Italian families, includes testing for 38 metabolic disorders and is strongly recommended. Additionally, transfontanellar ultrasound, a non-invasive and widely accessible technique, is advised for detecting potential brain malformations. The utility and timing of magnetic resonance imaging (MRI) remain subjects of debate, as MRI findings have identified anomalies in the brainstem and cerebellar hypoplasia in a limited number of cases. However, current evidence suggests that brain imaging findings offer limited utility in guiding patient management. Early initiation of a personalized rehabilitation program is critical; therefore, neurological and psychiatric evaluations should be conducted within the first few weeks following diagnosis or as promptly as feasible [30].

When analyzing the clinical symptoms of patients with CdCS, each symptom does not create a distinct phenotype. However, when combined with the characteristic cry associated with the condition, this can lead to a suspected diagnosis at birth. A karyotype analysis of the peripheral blood can provide confirmation of the diagnosis. For moderate situations where the diagnosis may be missed, especially in senior patients, the clinical presentation (particularly the aberrant voice) and psychomotor delay will prompt cytogenetic and molecular cytogenetic studies [9].

#### 3.1.3. Prognosis

According to Niebuhr, children with CdCS have a relatively high mortality rate, reaching up to 90% within the first year, but morbidity is modest [9].

It is scientifically documented that early intervention in children with neurodevelopmental and other disorders provides significant individual and social long-term benefits. Children develop numerous skills and improve cognitive and linguistic areas thanks to the brain’s neuroplasticity, which is significantly enhanced during development by providing a variety of stimuli. Consequently, a range of targeted supportive interventions during the early years of a child’s life plays a critical role in shaping their social, emotional, and cognitive development, while also enhancing their potential for successful integration. Recent research shows that the early inclusion of children with CdCS in rehabilitation programs improves their chances of integrating into the community. Individualized SLT interventions improve children with CdCS’s psychomotor development and communicative and swallowing flexibility, which contribute significantly to their independent living and, as a result, their social adaptation [32].

### 3.2. Speech, Language, and Swallowing Interventions

The speech and language evaluation results showed poor expressive, receptive, and pre-linguistic delays. Difficulties in expressive communication (the ability to express oneself) are very common in children with this neurodevelopmental disorder. In addition, a large percentage of individuals have little or no speech. It is evident that expressive language difficulties are associated with physical abnormalities in the larynx (vocal cords) and the delayed development of motor skills. However, the most important cause is a brain dysfunction towards the initiation of speech. It is estimated that about 3 out of 10 people use single words to communicate. Many children and adults with CdCS compensate for their verbal communication difficulties by using a range of gestures or signs to support their verbal communication. It is also found from studies that, while children and adults with CdCS are highly motivated to communicate, poor motor control and orofacial coordination difficulties can lead to a variety of inaccuracies regarding the articulation of verbal communication [23].

Due to the significant expressive language deficits, many children with CdCS benefit from augmentative and alternative communication (AAC) systems. These methods, which include sign language, picture exchange communication systems (PECSs), and communication devices, allow individuals to express their needs and emotions more effectively, compensating for verbal limitations [28].

Children often exhibit significant deficits in learning abilities, language development, and academic achievement. Their receptive language skills are generally stronger than their expressive skills, although both are delayed. There are omissions, substitutions, and assimilations to vowels, while speech is poor, with overlapping vowels, the limited use of syllables, and word distortions. The average length of utterances usually does not exceed two to three words during vocabulary development. For this reason, the case of this syndrome in the field of language needs to be investigated more thoroughly [27].

The science of speech therapy can help individuals with CdCS improve their communication skills. A speech pathologist (SP) can work with the individual to address speech and language difficulties such as articulation, vocabulary development, and social communication skills. The SP may also work with the individual’s family members and caregivers to provide support and strategies for communication at home and in the community [33].

Overall, speech therapy can play an important role in supporting individuals with CdCS in achieving their communication goals, overcoming their swallowing problems, and improving their quality of life.

### 3.3. Management of Swallowing Difficulties

Mild swallowing difficulties are mostly due to low muscle tone in the face and mouth. Swallowing problems mainly affect the oral and pharyngeal phases. Dealing with both developmental and motor abnormalities can make managing swallowing challenging. However, early symptom diagnosis can help tailor treatment to promote proper swallowing and prevent further issues. The treatment plan can include strategies to improve swallowing safety and efficiency.

The initial approach to symptom management should be based on a comprehensive swallowing assessment by a qualified speech and language therapist. This SLT report includes an assessment of muscle tone, anatomical and functional integrity, reflexes, and coordination of the structures involved in feeding and swallowing.

Furthermore, cognitive and motor skills, as well as the motor programming of the swallowing structures, are assessed [39].

It is important to provide constant visual cues to promote safe swallowing. Taking the correct position when feeding can significantly impact the management of dysphagia. Improved swallowing performance can result from the correct seat and head positioning. Implementing individualized feeding strategies can help people with CdCS manage dysphagia more effectively.

The treatment plan can include oral–motor exercises to improve oral–motor strength, coordination, and control of anatomical structures. These exercises help children develop clear articulation and enhance the necessary muscular control for safe swallowing. To promote effective swallowing, it is advisable to employ compensatory techniques such as adjusting head and body posture, making dietary adjustments including modifying the consistency, temperature, and size of the food, and adapting feeding and hydration routines using specialized equipment such as special utensils or cups. Specifically, these strategies facilitate solid and liquid food management and contribute to safer swallowing. Additionally, it is recommended to use adapted utensils, modify food composition, or alter the feeding environment to minimize distractions (Table 4).

Individuals and caregivers can acquire knowledge of specific swallowing maneuvers and strategies to facilitate improved swallowing. Techniques such as chin tucks and head placement may be employed to enhance swallowing safety. Collaboration with a team of medical specialists, including dietitians, gastroenterologists, and pediatricians, is essential. Their involvement enables the monitoring of growth, the provision of guidance on nutritional requirements, and the management of gastrointestinal issues associated with dysphagia [43,44].

A summarized table for speech, language, and swallowing deficits in CdCS, integrating cognitive deficits as a critical component influencing language processing with appropriate therapeutic suggestions, as synthesized from the references, can be found in Table 3.

**Table 3:** Summary of speech, language, swallowing, and cognitive deficits in CdCS with associated therapeutic interventions.

Deficit Type	Description	Contributing Factors	Impact/Challenges	Therapeutic Findings	References
Speech Deficits	- Significant delays in speech production - Many remain nonverbal or have limited verbal output - Articulation difficulties and limited vocabulary	- Hypotonia affecting speech muscles - Intellectual disability - Oral–motor dysfunction - Poor phonological memory	- Delayed onset of spoken words (2.5–5 years) - Reliance on alternative communication systems - Poor articulation, limited vocabulary, and distorted speech	- Use of augmentative and alternative communication (AAC) tools like PECS or sign language - Speech therapy focusing on articulation, motor coordination, and phonology	[1,7,9,14,21,23,25,27]
Expressive Language	- Severely impaired compared to receptive language - Difficulty articulating thoughts and forming complex sentences	- Motor and cognitive constraints - Hypotonia - Deficits in working memory	- Difficulty achieving functional speech - Limited length of utterances (2–3 words) - Impaired communication effectiveness	- Tailored language interventions - Repetition, multi-sensory learning approaches - Supportive strategies for vocabulary development	[10,12,20,21,23,26,27, 30,32,33,34,35,36,37, 38,40,41,42,45,47,49]
Receptive Language	- Delays in comprehension of complex instructions and abstract concepts - Better than expressive skills	- Attention deficits - Poor phonological and syntactic memory	- Difficulty following multi-step directions - Limited understanding of abstract language	- Use of visual aids and step-by-step instructions - Simplified language and structured teaching methods	[7,9,14,15,23,25,28,31,32,33,34,36,40,46,48, 50]
Swallowing Deficits	- Feeding difficulties and unsafe swallowing - Gastroesophageal reflux - Poor saliva management	- Hypotonia of feeding muscles - Oral–motor dysfunction - Poor tongue control and delayed swallowing reflex	- Risk of malnutrition, dehydration, and aspiration - Challenges in oral and pharyngeal phases of swallowing	- Oral–motor exercises - Compensatory techniques (e.g., posture adjustments, food modification) - Collaboration with dietitians and medical specialists	[4,25,27,28,31,33,34,39,42,43,44,49,51]
Cognitive Deficits	- Impaired executive function and memory - Poor attention regulation and working memory	- Genetic deletions affecting brain development - Early attentional deficits - Limited cognitive flexibility and inhibitory control	- Challenges in problem-solving and adapting to new routines - Impact on language acquisition and multi-step task completion	- Memory training exercises - Visual and multi-sensory learning methods - Strategies to improve executive function and focus	[7,9,23,28,29,31,50,52,53]

## 4. Discussion

We examined the current literature regarding CdCS to comprehensively understand its clinical presentation, genetic basis, and the challenges associated with diagnosis and management. This review aims to synthesize existing knowledge to identify research gaps and explore opportunities for improved therapeutic interventions and support systems. By examining the genetic and phenotypic variability among affected individuals, we seek to provide insights that may guide personalized care approaches and inform future studies on the molecular mechanisms underlying the syndrome. Additionally, this overview highlights the importance of multidisciplinary care teams and early intervention programs in enhancing the quality of life for individuals with CdCS and their families.

With an emphasis on SLT as a therapeutic strategy, this study attempts to thoroughly examine the clinical picture of CdCS and the difficulties that are connected with it. It looks at the methods used in SLT to address the range of communication, feeding and swallowing difficulties, oral–motor impairments, and social communication challenges seen in CdCS. In addition to providing insights into existing procedures and possible therapy breakthroughs that could improve functional results and quality of life for patients with CdCS, the research highlights the significance of SLT in managing these complex issues.

The role of an SP in the intervention and therapy of children with CdCS is crucial due to the significant communication challenges that these children face. Speech and language impairments are among the most pronounced features of CdCS, often resulting in limited expressive language, poor articulation, and difficulty acquiring functional verbal skills [23]. Effective early intervention by SP can improve the quality of life for children with CdCS, aiding both language development and overall cognitive growth.

Communication difficulties, such as speech perception and performance, are common among individuals with CdCS. These challenges may be linked to physical abnormalities in the vocal cords and delayed motor skill development. Individuals with CdCS often rely on nonverbal communication and imitation to compensate for these difficulties, as poor motor control and swallowing issues can result in inadequate verbal communication and physical development. Early intervention is vital in addressing the pervasive speech and language delays seen in CdCS. By identifying and working on communication difficulties at an early age, SPs can enhance a child’s capacity to develop language skills, even in the context of severe intellectual and physical limitations. Intervention during the early developmental stages capitalizes on neuroplasticity, where the brain is more adaptable to learning new skills, including language acquisition [27]. Children with CdCS who receive early intervention may experience improved outcomes in expressive and receptive communication, which are key components of their overall cognitive development.

SPs also contribute in supporting the development of alternative and augmentative communication (AAC) systems for children with severe expressive language deficits. Given that many children with CdCS remain nonverbal or have minimal speech capabilities, AAC methods may include sign language, picture exchange communication systems (PECSs), and electronic communication devices that can offer practical alternatives for communication. By facilitating the use of AAC, SLTs enable children to express needs, engage socially, and improve their autonomy, significantly enhancing their quality of life. Children with CdCS typically show a more significant impairment in expressive language than in receptive language [23]. SLTs focus on improving articulation, phonological skills, and vocabulary development. Therapy may include exercises to strengthen the oral–motor functions necessary for speech production, mainly because low muscle tone (hypotonia) is common in CdCS and can impact speech clarity [9]. Addressing these physical barriers through targeted therapy improves speech intelligibility and boosts confidence and participation in social interactions.

In addition, SPs employ strategies to encourage expressive language development through play-based activities and structured language practice. These activities are tailored to the individual’s cognitive abilities, using methods such as repetition and positive reinforcement to support language learning. Children with CdCS can improve their expressive capabilities through consistent and focused intervention, even if progress is slow.

SLT can help individuals with CdCS and improve their communication skills. An SP can address issues such as articulation, vocabulary development, social communication skills, and swallowing efficiency. The therapist may also collaborate with the individual’s family and caregivers to provide support and strategies for communication and swallowing at home and in the community. The consistent application of these methods, with two weekly sessions for at least two years, has been demonstrated to enhance communication skills in children with CdCS. Treatment for these disorders is continuous and adjusted based on the child’s developmental needs. The early detection of speech and swallowing difficulties allows therapists to implement strategies that harness neuroplasticity during critical developmental windows. Pediatric neurology follow-ups and continuous reassessments by therapists provide feedback to optimize treatment outcomes. Ongoing research aims to enhance the effectiveness of interventions by speech and language therapists.

Though receptive language skills are generally stronger than expressive abilities in children with CdCS, they are still impaired, and children often struggle with understanding complex instructions or abstract language concepts [9]. SPs enhance comprehension by simplifying language input, using visual aids, and employing repetitive and consistent language structures. This is essential in helping children process spoken language, follow instructions, and engage meaningfully in educational and social settings.

Therapists often integrate AAC methods into receptive language therapy, using visual symbols, gestures, or pictures to bridge gaps in comprehension (Table 4). This multi-modal approach supports learning new concepts and words, ensuring that children can process and respond to their environment more effectively [27].

Another key aspect of SLT intervention in CdCS is the collaboration between therapists, families, and educators. SLTs work closely with families to create home-based communication strategies that reinforce therapy goals, ensuring consistency across different environments. Parents and caregivers are educated on using AAC devices or communication tools effectively, fostering a supportive environment for language development.

Moreover, SLTs often coordinate with other professionals, such as occupational therapists and educators, to address the broader developmental needs of the child. For instance, integrating speech and language goals into a child’s adapted teaching plan allows for a more comprehensive approach to learning and development in school settings [7].

### 4.1. A Holistic Therapeutic Framework for Managing Speech, Language, and Swallowing Challenges in CdCS

The genetic mutation associated with CdCS results in early brain damage, which manifests from fetal development and significantly impacts motor, cognitive, and communication abilities. These challenges persist throughout the individual’s life and necessitate a proactive, multidisciplinary approach [23]. Early intervention is critical for improving communication and language abilities in children with CdCS [37]. A comprehensive speech therapy assessment can help identify the child’s communication profile, cognitive strengths, motor abilities, and medical challenges. Tailored therapy programs focusing on cognitive, linguistic, and swallowing skills, while adaptable to the child’s progress, are essential to achieving developmental goals.

### 4.2. Limitations

The studies reviewed may have only involved a small number of subjects due to the rarity of *CdCS*, which could limit the findings’ statistical power and generalizability. Additionally, the severity of cognitive, motor, and speech difficulties in children with *CdCS* can vary greatly. It may be challenging to extrapolate findings to all people with the condition due to this diversity. Therapeutic results may be significantly influenced by the level of parental or caregiver involvement, which can differ greatly amongst participants and impact the consistency of the outcomes. The research was conducted using data obtained from limited cases due to the rarity of the syndrome on tools or methods that are not widely accessible, making it challenging to replicate in environments with limited resources.

### 4.3. Ethical Considerations

The experimental design may be limited by the population’s vulnerability, such as the inability to include control groups not receiving any treatment.

### 4.4. Scientific Gap—Future Research

Although this guide and previous research offer useful information on swallowing and communication therapy for kids with CdCS, some significant gaps could be filled in the future. The lack of longitudinal research to confirm the efficacy of intervention approaches is the main finding of this study. There is a need for the generalization of assistive technology use and its application based on individualized protocols, culturally and linguistically tailored to the situation, and the integration of holistic multidisciplinary interventions to address communication and swallowing challenges.

## 5. Conclusions

The involvement of a speech and language therapist is indispensable in the therapeutic and intervention processes for children with CdCS. Early targeted interventions focusing on expressive and receptive language skills can significantly improve communication, promoting greater independence and social interaction. Additionally, the use of AAC systems and the collaboration between SP, family, and other professionals are essential in providing a holistic, supportive framework for the child’s communication development. Although children with CdCS face lifelong challenges in language and communication, the role of the SP is instrumental in maximizing their potential and improving their overall quality of life. The SP regularly reassesses dysphagia and other speech and language abnormalities as part of an ongoing process to monitor the progress of individuals with CdCS and adjust according to their evolving needs. Regular evaluations can help identify problems and address any new concerns that arise. Involving parents in s child’s therapy with a speech pathologist can significantly contribute to treating the child’s issues holistically.

## Figures and Tables

**Table 2:** Summary of clinical characteristics of Cri du Chat Syndrome.

Category	Clinical Feature	Frequency/Prevalence	Comments
Craniofacial Features	High-pitched, cat-like cry	95.9%	Often present at birth, diagnostic indicator
	Microcephaly	Common	More evident with age
	Epicanthal folds and telecanthus	90.2% and 81.4%	Common facial dysmorphisms
	Low-set ears and micrognathia	69.8% and 96.7%	Additional craniofacial dysmorphisms
	Long, narrow face and prominent supra-orbital arch	70.8% and 31.0%	Develop with age
	Dental malocclusion (open bite)	75.0%	Common dental anomaly
	Strabismus or divergent strabismus	Increased risk (44.7%)	Higher prevalence in patients with CdCS
Cognitive and Developmental	Severe mental and psychomotor delay	Common	Varies with the size of the deletion
	Severe delay in language expression compared to comprehension.	Characteristic	Progress in verbal development is slow
	Behavioral issues (self-injury and aggression)	Common	Include hypersensitivity and compulsive attachment to objects
Neurological	Seizures	Rare	Brainstem atrophy observed via MRI
	Atrophy of pons, cerebellum, and white matter	MRI findings	Not typical, with rare cases of arachnoid cyst reported
Physical Anomalies	Low birth weight	2614 g (mean)	Neonatal feature, along with perinatal asphyxia
	Feeding difficulties (dysphagia)	Common	Major issue for overall development
	Cardiac defects (VSD, ASD, and PDA)	Present	Include more severe defects like tetralogy of Fallot
	Hypotonia (early years) and hypertonia (later)	Progression	Muscle tone changes with age
	Attention deficit disorder with hyperactivity	Present (~50%)	Sometimes coexists with aggressiveness
Other Features	Cryptorchidism, rare in adolescents	Occasional at birth	Normal sexual development
	Myopia and cataracts	Reported	Common ocular anomalies
	Gentle and affectionate personality	Common	Autistic features and impaired social interaction are rare

**Table 4:** Key focus areas in holistic intervention approaches.

Focus Area	Intervention Strategy	Objective
Multi-Sensory Approaches	Use of visual aids, gestures, and sensory cues.	Enhance communication, learning, and feeding skills.
Oral–Motor Exercises	Strengthen articulatory mobility, muscle tone, and coordination.	Improve speech production, chewing, and swallowing safety.
Simplified Language	Implement simplified structures and frequent repetition.	Support expressive and perceptual language development.
Optical Hints	Use visual cues such as symbols, images, and social stories.	Promote knowledge generalization and social adaptation.
Robotics in Therapy	Utilize interactive robots to deliver speech, language, and swallowing prompts.	Provide engaging therapy, enhance motivation, and support SPs in addressing complex therapy goals.
Augmentative Communication	Employ AAC systems (e.g., communication boards, sign language).	Bridge communication gaps and support early language development.
Music Therapy	Engage with musical instruments and rhythm-based activities.	Improve coordination, emotional expression, and cognitive functions.

## References

[1] Sigafoos J., O’Reilly M.F., Lancioni G.E. (2009). Cri-Du-Chat. Dev. Neurorehabilit..

[2] Lejeune J., Lafourcade J., Berger R., Vialatte J., Boeswillwald M., Seringe P., Turpin R. (1963). 3 cases of partial deletion of the short arm of a 5 chromosome. Comptes Rendus Hebd. Séances Académie Sci..

[3] Corrêa T., Feltes B.C., Riegel M. (2019). Integrated Analysis of the Critical Region 5p15.3-P15.2 Associated with Cri-Du-Chat Syndrome. Genet. Mol. Biol..

[4] Holland P., Wildhagen M., Istre M., Reiakvam O.M., Dahl J.A., Søraas A. (2022). Cri Du Chat Syndrome Patients Have DNA Methylation Changes in Genes Linked to Symptoms of the Disease. Clin. Epigenet..

[5] Overhauser J., Huang X., Gersh M., Wilson W., Mcmahon J., Bengtsson U., Rojas K., Meyer M., Wasmuth J.J. (1994). Molecular and Phenotypic Mapping of the Short Arm of Chromosome 5: Sublocalization of the Critical Region for the Cri-Du-Chat Syndrome. Hum. Mol. Genet..

[6] Gersh M., Goodart S.A., Pasztor L.M., Harris D.J., Weiss L., Overhauser J. (1995). Evidence for a Distinct Region Causing a Cat-like Cry in Patients with 5p Deletions. Am. J. Hum. Genet..

[7] Cornish K., Bramble D. (2002). Cri Du Chat Syndrome: Genotype-Phenotype Correlations and Recommendations for Clinical Management. Dev. Med. Child Neurol..

[8] Higurashi M., Oda M., Iijima K., Iijima S., Takeshita T., Watanabe N., Yoneyama K. (1990). Livebirth Prevalence and Follow-up of Malformation Syndromes in 27,472 Newborns. Brain Dev..

[9] Mainardi P.C., Pastore G., Castronovo C., Godi M., Guala A., Tamiazzo S., Provera S., Pierluigi M., Bricarelli F.D. (2006). The Natural History of Cri Du Chat Syndrome. A Report from the Italian Register. Eur. J. Med. Genet..

[10] Pizzamiglio M.R., Volpe C., Piccardi L., Pizzamiglio M.R., Volpe C., Piccardi L. (2013). A Longitudinal Study in Atypical Cri-Du Chat Profile: A Single Case Report. Case Rep. Clin. Med..

[11] Corcuera-Flores J.R., Casttellanos-Cosano L., Torres-Lagares D., Serrera-Figallo M.Á., Rodríguez-Caballero Á.N.G.E.L.A., Machuca-Portillo G. (2016). A Systematic Review of the Oral and Craniofacial Manifestations of Cri Du Chat Syndrome. Clin. Anat..

[12] Bagchi N.R., Bhanja S. (2015). Cri Du Chat Syndrome: A Case Report with Recurrent Pneumonia and Chronic Stridor. Int. J. Pediatr..

[13] Niebuhr E. (1978). The Cri Du Chat Syndrome: Epidemiology, Cytogenetics, and Clinical Features. Hum. Genet..

[14] Rodríguez-Caballero Á., Torres-Lagares D., Rodríguez-Pérez A., Serrera-Figallo M.Á., Hernández-Guisado J.M., Machuca-Portillo G. (2010). Cri Du Chat Syndrome: A Critical Review. Med. Oral Patol. Oral Cirugia Bucal.

[15] Ajitkumar A., Jamil R.T., Mathai J.K. (2022). Cri Du Chat Syndrome. Brenner’s Encyclopedia of Genetics.

[16] Mainardi P.C., Perfumo C., Calì A., Coucourde G., Pastore G., Cavani S., Zara F., Overhauser J., Pierluigi M., Bricarelli F.D. (2001). Clinical and Molecular Characterisation of 80 Patients with 5p Deletion: Genotype-Phenotype Correlation. J. Med. Genet..

[17] Lefranc V., de Luca A., Hankard R. (2016). Protein-Energy Malnutrition Is Frequent and Precocious in Children with Cri Du Chat Syndrome. Am. J. Med. Genet. Part A.

[18] Cerruti Mainardi P. (2006). Cri Du Chat Syndrome. Orphanet J. Rare Dis..

[19] Pizzamiglio M.R., Nasti M., Piccardi L., Vitturini C., Morelli D., Guariglia C. (2008). Visual-Motor Coordination Computerized Training Improves the Visuo-Spatial Performance in a Child Affected by Cri-Du-Chat Syndrome. Int. J. Rehabil. Res..

[20] Sweeney S. (2012). Cri Du Chat Syndrome: Case Presentation and Review. J. Behav. Optom..

[21] Zhang X., Snijders A., Segraves R., Zhang X., Niebuhr A., Albertson D., Yang H., Gray J., Niebuhr E., Bolund L. (2005). High-resolution mapping of genotype–phenotype relationships in cri du chat syndrome using array comparative genomic hybridization. Am. J. Hum. Genet..

[22] Chistol M., Turcu C., Danubianu M. (2023). Autism Assistant: A Platform for Autism Home-Based Therapeutic Intervention. IEEE Access.

[23] Cornish K.M., Pigram J. (1996). Developmental and Behavioural Characteristics of Cri Du Chat Syndrome. Arch. Dis. Child..

[24] Harris B.N., Kuhn M., Evangelista L., Davis S., Quimby A., Parmar S., Fernandes R. (2023). Speech and Swallow Therapy. Complex Head and Neck Microvascular Surgery: Comprehensive Management and Perioperative Care.

[25] Tullu M.S., Muranjan M.N., Sharma S.V., Sahu D.R., Swami S.R., Deshmukh C.T., Bharucha B.A. (1998). Cri-Du-Chat Syndrome: Clinical Profile and Prenatal Diagnosis. J. Postgrad. Med..

[26] Cornish K.M., Munir F. (1998). Receptive and Expressive Language Skills in Children with Cri-Du-Chat Syndrome. J. Commun. Disord..

[27] Kristoffersen K.E. (2008). Speech and Language Development in Cri Du Chat Syndrome: A Critical Review. Clin. Linguist. Phon..

[28] Wu Q., Niebuhr E., Yang H., Hansen L. (2005). Determination of the ‘critical region’ for cat-like cry of Cri-du-chat syndrome and analysis of candidate genes by quantitative PCR. Eur. J. Hum. Genet..

[29] Cornish K.M. (1996). The Neuropsychological Profile of Cri Du Chat Syndrome without Significant Learning Disability. Dev. Med. Child Neurol..

[30] Liverani M.E., Spano A., Danesino C., Malacarne M., Cavani S., Spunton M., Guala A. (2019). Children and Adults Affected by Cri Du Chat Syndrome: Care’s Recommendations. Pediatr. Rep..

[31] Guala A., Spunton M., Kalantari S., Kennerknecht I., Danesino C. (2017). Neoplasia in Cri Du Chat Syndrome from Italian and German Databases. Case Rep. Genet..

[32] Lokmanoğlu B.N.Y., Mutlu A., Livanelioğlu A., Haliloğlu G. (2021). The General Movements Assessment and Effects of an Early Intervention in an Infant with Cri Du Chat Syndrome: A Case Report. Turk. J. Pediatr..

[33] Virbalas J.M., Palma G., Tan M. (2012). Obstacles to Communication in Children with Cri Du Chat Syndrome. J. Voice.

[34] Honjo R.S., Mello C.B., Pimenta L.S.E., Nuñes-Vaca E.C., Benedetto L.M., Khoury R.B.F., Befi-Lopes D.M., Kim C.A. (2018). Cri Du Chat Syndrome: Characteristics of 73 Brazilian Patients. J. Intellect. Disabil. Res..

[35] Butt A.K., Zubair R., Rathore F.A. (2022). The Role of Augmentative and Alternative Communication in Speech and Language Therapy: A Mini Review. J. Pak. Med. Assoc..

[36] Sofologi M., Papatzikis E., Kougioumtzis G., Kosmidou E., Klitsioti A., Droutme A., Sourbi A.A., Chrisostomou D., Efstratopoulou M. (2022). Effectiveness of Musical Training on Reading Comprehension in Elementary School Children. Is There an Associative Cognitive Benefit?. Front. Educ..

[37] Giridhar S. (2020). Cri-Du-Chat Syndrome: A Case Report. Int. J. Health Sci. Res..

[38] Taheri A., Meghdari A., Alemi M., Pouretemad H., Poorgoldooz P., Roohbakhsh M. (2016). Social Robots and Teaching Music to Autistic Children: Myth or Reality?. Lect. Notes Comput. Sci..

[39] Triggs J., Pandolfino J. (2019). Recent Advances in Dysphagia Management. F1000Research.

[40] Cornish K.M., Bramble D., Munir F., Pigram J. (1999). Cognitive Functioning in Children with Typical Cri Du Chat (5p-) Syndrome. Dev. Med. Child Neurol..

[41] Levy B., Dunn T.M., Kern J.H., Hirschhorn K., Kardon N.B. (2002). Delineation of the Dup5q Phenotype by Molecular Cytogenetic Analysis in a Patient with Dup5q/Del 5p (Cri Du Chat). Am. J. Med. Genet..

[42] Marinescu R.C., Johnson E.I., Grady D., Chen X.N., Overhauser J. (1999). FISH Analysis of Terminal Deletions in Patients Diagnosed with Cri-Du-Chat Syndrome. Clin. Genet..

[43] Lass N.J., Pannbacker M. (2008). The Application of Evidence-Based Practice to Nonspeech Oral Motor Treatments. Lang. Speech Hear. Serv. Sch..

[44] Nakao-Kato M., Rathore F.A. (2023). An Overview Of The Management And Rehabilitation Of Dysphagia. J. Pak. Med. Assoc..

[45] Espirito Santo L.D., Moreira L.M.A., Riegel M. (2016). Cri-Du-Chat Syndrome: Clinical Profile and Chromosomal Microarray Analysis in Six Patients. BioMed Res. Int..

[46] Buggenhout G., Pijkels E., Holvoet M., Schaap C., Hamel B., Fryns J. (2000). Cri Du Chat Syndrome: Changing Phenotype in Older Patients. Am. J. Med. Genet..

[47] Castillo J.C., Álvarez-Fernández D., Alonso-Martín F., Marques-Villarroya S., Salichs M.A. (2018). Social Robotics in Therapy of Apraxia of Speech. J. Healthc. Eng..

[48] Villa R., Fergnani V.G.C., Silipigni R., Guerneri S., Cinnante C., Guala A., Danesino C., Scola E., Conte G., Fumagalli M. (2020). Structural Brain Anomalies in Cri-Du-Chat Syndrome: MRI Findings in 14 Patients and Possible Genotype-Phenotype Correlations. Eur. J. Paediatr. Neurol. Off. J. Eur. Paediatr. Neurol. Soc..

[49] Sanders C.D., Leigh M.W., Chao K.C., Weck K.E., King I., Wolf W.E., Campbell D.J., Knowles M.R., Zariwala M.A., Shapiro A.J. (2018). The Prevalence of the Defining Features of Primary Ciliary Dyskinesia within a Cri Du Chat Syndrome Cohort. Pediatr. Pulmonol..

[50] Bel-Fenellós C., Biencinto-López C., Sáenz-Rico B., Hernández A., Sandoval-Talamantes A.K., Tenorio-Castaño J., Lapunzina P., Nevado J. (2023). Cognitive-Behavioral Profile in Pediatric Patients with Syndrome 5p-; Genotype-Phenotype Correlationships. Genes.

[51] Wapner R.J., Babiarz J.E., Levy B., Stosic M., Zimmermann B., Sigurjonsson S., Wayham N., Ryan A., Banjevic M., Lacroute P. (2015). Expanding the Scope of Noninvasive Prenatal Testing: Detection of Fetal Microdeletion Syndromes. Am. J. Obstet. Gynecol..

[52] Ye Y., Luo Y., Qian Y., Xu C., Jin F. (2011). Cri Du Chat Syndrome after Preimplantation Genetic Diagnosis for Reciprocal Translocation. Fertil. Steril..

[53] Georgieva-Tsaneva G., Andreeva A., Tsvetkova P., Lekova A., Simonska M., Stancheva-Popkostadinova V., Dimitrov G., Rasheva-Yordanova K., Kostadinova I. (2023). Exploring the Potential of Social Robots for Speech and Language Therapy: A Review and Analysis of Interactive Scenarios. Machines.

